# Impact of the 2020 pandemic of COVID-19 on Families with School-aged Children in the United States: Roles of Income Level and Race

**DOI:** 10.1177/0192513X21994153

**Published:** 2022-03

**Authors:** Cliff Yung-Chi Chen, Elena Byrne, Tanya Vélez

**Affiliations:** 1 Educational and Community Programs, Queens College of the City University of New York, Flushing, NY, USA

**Keywords:** COVID-19, pandemic, school closure, work & family, parenting

## Abstract

This study examined the experiences of families with school-aged children during the first three months of the 2020 pandemic of COVID-19 in the United States, while focusing on the roles of income level and race/ethnicity in their experiences. Two hundred and twenty-three parents of school-aged children participated in this study by completing an online survey. The results revealed that low-income and lower-middle class parents, as well as parents of color, experienced more instrumental and financial hardships due to the pandemic, when compared to their higher income, White counterparts. In contrast, parents with higher income and White parents were more likely to feel stressed over structuring home learning environments and planning educational and physical activities at home for their children. The overall findings suggest that family income level and race/ethnicity play a significant role in the lives of families coping with a variety of challenges due to the pandemic.

On March 13, 2020, the United States declared a national emergency over the outbreak of Coronavirus Disease (COVID-19) as the novel, highly transmissible Coronavirus (SARS-CoV-2) started rapidly spreading across the country. Due to the nature of the virus, by the end of March 2020, more than half of the U.S. population was ordered to stay home under shelter-in-place orders issued by many states and cities across the county in an attempt to minimize close contact between people in order to reduce the spread of the virus. As a result, more than 124,000 schools in the United States were closed, affecting at least 55 million students (Education Week, 2020). Although some cities/states gradually lifted their shelter-in-place orders and prepared for reopening in phases in late May or early June of 2020, the majority of schools remained closed for the rest of the 2019–2020 school year.

A pandemic outbreak can have a pervasive impact on social order and the economy (Qui et al., 2016–2017). For instance, schooling and the economy were significantly disrupted during the Ebola pandemic in 2013 and 2015 in West Africa in order to contain the virus (Nabarro & Wannous, 2016). During the H1N1 outbreak in 2009, large scale school dismissals/closures were found to lead to a greater reduction rate of infected cases (German et al., 2019). Araz et al. (2013) found that preventive closing was best to decrease unnecessary infection; however, it is important to consider issues of distance learning, school meals, and dealing with pandemic waves. With the United States being the hardest-hit country by the COVID-19 pandemic in terms of numbers of COVID-19 infected cases and deaths (Johns Hopkins University and Medicine, 2020), many families with school-aged children experience various challenges due to the outbreak and subsequent lockdowns and life disruptions.

In addition to worries and anxieties related to the COVID-19 outbreak, the economic situation has suddenly worsened with unprecedented rising levels of unemployment in the first three months of COVID-19 in the U.S. (Kochhar, 2018), resulting in financial hardship for many families. Moreover, external support by other family members may be disrupted and social support systems may fade away due to social distancing measures. Many parents, while working from home, have to take care of their children with restricted caregiver resources (e.g., grandparents, daycare settings), as well as support their children’s education through home schooling or remote learning provided by their schools (Fegert et al., 2020). This has suddenly created a lot of challenges and put a lot of pressure on parents of schooling children. This study explored the consequences and problems associated with the COVID-19 outbreak as experienced by families with school-aged children during the acute phase of COVID-19 in the U.S.

Past research has suggested that a pandemic may affect different demographics differently, leading to social, economic, and health disparities (Kumar et al., 2012). As times of crisis often reinforce and exacerbate these disparities because resources are limited and people are fearful, traditionally minoritized and marginalized populations (e.g., racial/ethnic minorities, low-income families, women) may encounter more challenges (Kantamneni, 2020). As part of the long history of racial bias and discrimination in the U.S., national disasters and crises have often become racialized and scapegoated minority groups are targeted and blamed (Chen et al., 2020). Evidently, racial discrimination against Asians and Asian Americans has increased significantly since the outbreak of the COVID-19 in the U.S. (e.g., Chen et al., 2020; Lee & Waters, 2020). According to a Pew Research Center survey, Black and Asian Americans have shared the experiences of racial discrimination amid the COVID-19 pandemic. Approximately 40% of Black and Asian Americans reported that since the COVID-19 outbreak people have acted as if they were uncomfortable around them due to their race or ethnicity (Ruiz et al., 2020).

Recent data from Centers for Disease Control and Prevention (CDC, 2020a) indicated that COVID-19 disease burden, including acquisition of illness, hospitalization, and mortality, is disproportionately higher among racial and ethnic groups (e.g., Black/African American, Latinx, American Indian or Alaska Native, and Asian American). Persistent systemic inequities, such as structural racism and discrimination, overcrowded housing, occupational segregation, and inadequate health access and utilization, play a large role in contributing to disparities in health outcomes among minoritized populations (cited in Brown et al., 2020; CDC, 2020b). Evidence has suggested that Black and Hispanic workers face much more economic and health insecurity as a result of the COVID-19 outbreak than White workers (Gould & Wilson, 2020). Being that racial and ethnic minority groups (e.g., Black and Latino people) are overrepresented in low-wage jobs and in jobs that cannot transition to remote work, they have been hit the hardest by stay-at home and other public health measures that put in place to control the spread of the virus. Recent data suggest that people of color and low-income families, when compared to their White and higher-income counterparts, have been affected much more by spiking unemployment and job insecurity (Gould & Wilson, 2020), as well as increased housing instability (Greens & McCargo, 2020), since the COVID-19 outbreak. These economic and social inequities may in turn place minoritized families at greater risk for increased stress and disparate outcomes during the COVID-19 pandemic.

School closures due to the COVID-19 outbreak have added extra challenges for parents of schooling children. Moreover, prolonged school closures can further exacerbate preexisting educational disparities. Reports have shown that lower-income parents are more concerned about their children potentially falling behind amid COVID-19 school closures than higher-income parents (Horowitz, 2020). While many schools have abruptly adopted remote learning to continue students’ schooling in response to the outbreak, students from lower-income households experience a “digital gap” due to the lack of reliable access to the Internet and other digital resources (e.g., computers) at home, potentially affecting their learning. Data reported by Pew Research Center further suggest that the digital gaps are particularly notable in Black and Hispanic households of low incomes (Auxier & Anderson, 2020), suggesting race and family income contribute to the complexity of widening educational inequities as a result of the COVID-19 outbreak.

According to Prime et al.’s (2020) conceptual framework, the impact of social disruption due to COVID-19 (e.g., job loss, financial hardship, social distancing, confinement) needs to be considered in the context of preexisting vulnerabilities in families (e.g., racism and marginalization, economic hardship, history of adversity). Although the current strain of the COVID-19 outbreak is unprecedented and affects all people globally, the impact of the virus can be rooted in historic and persistent social, economic, and educational disparities, resulting in greater vulnerabilities and difficulties in people of color and low-income families. This study further examined the roles of income level and race/ethnicity in families’ experiences coping with the COVID-19 pandemic, focusing on those with school-aged children. We expected to observe greater social, economic, and educational challenges and problems experienced by families of color and low-income households.

## Method

### Procedure

After obtaining permission from the university institutional review board in early April of 2020, we used a variety of online recruitment methods, including online postings on social media (e.g., Facebook and Twitter), discussion forums (e.g., Reddit), websites (e.g., Psychological Research on the Net), and emails, to recruit parents of school-aged children (PreK–12th grade) in the United States as our research participants. Parents completed an online survey via the SurveyMonkey platform. Participants were given an opportunity to enter a drawing for one of ten $15 gift cards. Data were collected during a period of 2.5 months between April 8, 2020, and June 15, 2020.

### Participants

The sample included 223 parents of school-aged children, with a mean age of 41.31 years (*SD* = 8.54). The vast majority (*n* = 202, 91%) of our participants were female, and nearly two-thirds were White/Caucasian (*n* =145, 65%). More than half of the participants resided in New York State (*n* = 127, 57%), with 25 other states (*n*s = 1-14) representing the rest of the sample. Half (*n* = 112, 50%) of the participants had two or more (*M* = 1.74, SD = 0.97) school-aged children across PreK-12 grade levels in their household. Eighteen percent of the participants had at least one child in preschool, 8% had at least one child in kindergarten, 43% had at least one child in elementary school, 26% had at least one child in middle school, and 29% had at least one child in high school. See Table 1 for the demographic information of our participants.

**Table 1.** Demographic Data of Sample.

	*n (%)*
Gender	
Female	202 (90.6)
Male	21 (9.4)
Race/Ethnicity	
Asian and Pacific Islander	16 (7.2)
American Indian and Alaska Native	3 (1.3)
Black/African American	16 (7.2)
Latin/Hispanic	33 (14.8)
White/Caucasian	145 (65.0)
Mixed/Biracial/Multicultural	7 (3.1)
Other	3 (1.3)
Family Structure	
Single-father household	3 (1.3)
Single-mother household	33 (14.8)
Two-parent home	181 (81.2)
Other	5 (2.2)
No response	1 (0.4)
Household Income (US Dollar)	
$25,000 or under	15 (6.7)
$25,001–$50,000	26 (11.7)
$50,001–$75,000	21 (9.4)
$75,001–$100,000	31 (13.9)
$100,001–$200,000	74 (33.2)
More than $200,000	46 (20.6)
Other	3 (1.3)
No response	7 (3.1)
Employment Status	
Full-time employed	130 (58.3)
Part-time employed	23 (10.3)
Self-employed	20 (9.0)
Homemaker	18 (8.1)
Out of work at the moment	23 (10.3)
Other	6 (2.7)
No response	1 (0.4)

### Measures

Demographic information (e.g., age, gender, race/ethnicity, household income, family structure, etc.) was collected.

#### Employment and job status

Two items were developed for this study to assess whether participants’ employment status had changed since the outbreak of the COVID-19 pandemic, as well as their spouse’s/partner’s status if applicable. In addition, participants were also asked to report the degree to which they and their spouse/partner had worked remotely due to the pandemic on a six-point scale, ranging from 1 (*0% of the time*) to 6 (*100% of the time*).

#### Learning at home

We developed an item for this study to assess whether parents had clear structure and routines for their children at home to guide their learning on a five-point scale, ranging from *strongly disagree* to *strongly agree*. An additional item was developed to assess the methods (e.g., distance learning through school, homeschooling, self-directed learning, etc.) that children used to continue their education at home if their schools had been closed due to the COVID-19 pandemic.

#### Consequences of school closure associated with the COVID-19 outbreak

We adapted the items used in a 2009 influenza A (H1N1) study conducted by the Centers for Disease Control and Prevention (CDC, 2010) to measure the consequences of school closure associated with the outbreak of COVID-19 in the United States. Sample consequences/problems included “missed work,” “child missed free or reduced-cost school meals,” and “lost pay or income.” We added four new items to the list (e.g., “arranged childcare,” “felt stressed over planning educational activities for your child,” etc.). Participants were allowed to check off all items that applied to them.

### Statistical Analysis

For the purpose of this study, we categorized participants into three income classes based on their reported annual household incomes (US Dollar): (a) low-income and lower-middle class (≤ $50,000), (b) middle class ($50,001–$100,000), and (c) upper-middle and high-income (**>** $100,000; Kochhar, 2018). Fifty-four percent of our participants were from upper-middle class or high-income households, 23% from middle-class households, and 18% lower-middle or low-income households.

Due to a small number of participants representing each of the racial/ethnic minority groups in our sample, we dummy coded race/ethnicity into 0 (White) and 1 (people of color; POC), with POC representing those who identified as Asian/Pacific Islander, American Indian/Alaska Native, Black/Africian American, Latinx/Hispanic, or mixed/biricial/multiracial.

Chi-square tests were conducted to compare group differences in measures of consequences/problems associated with the COVID-19 pandemic by income class and race/ethnicity. We performed all statistical analysis using IBM SPSS version 26. Cramer’s *V (*φ*_c_)* values were calculated to determine the effect sizes of chi-squared tests, taking the degree of freedom into consideration (Cohen, 1988).

## Results

### Preliminary Data Analysis

Income class was associated with family structure, χ^2^(6, *N*=212) = 61.56, *p*<.001, φ_*c*_=.38 (large effect size). There was a higher rate of single-parent households within the low-income and lower-middle class (62.5%) than those in the middle class (19.2%), as well as the upper-middle and high-income class (3.3%). There was a moderate relationship between income class and race/ethnicity, χ^2^(2, *N*=213) = 14.47, *p*=.001, φ_*c*_=.26 (median effect size); White families were more likely to be in the higher income class than their POC counterparts. Families of color represented 56% of the low-income and lower-middle class, 42% of the middle class, and 25% of the upper-middle and high-income class in this sample.

### Impact of the COVID-19 Pandemic

#### Employment status change

**Figure 1.**
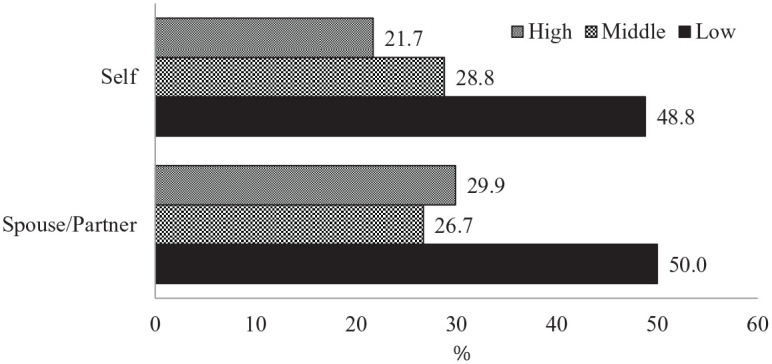
Employment status had changed by income level. *Note*. Low = low-income and lower-middle class (≤ $50,000); Middle = middle class ($50,001–$100,000); High = upper-middle and high-income class (>$100,000).

**Figure 2.**
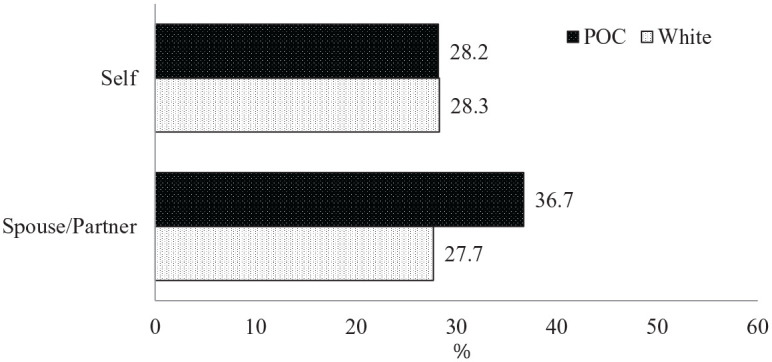
Employment status had changed by race/ethnicity. *Note*. POC = people of color.

More than a quarter (*n* = 63, 28.3%) of the participants reported that their employment status had changed since the outbreak of the COVID-19. These changes primarily included reduced work hours and reduced pay, furlough, and loss of employment, which tended to have adverse financial effects. Household income level was associated with employment status change, χ^2^(2, *N*=213) = 10.99, *p*=.004, φ_*c*_=.23 (medium effect size). Individuals from low-income and lower-middle class households reported the highest rate of employment status change (48.8%), followed by the middle class (28.8%), and the upper-middle class and high-income households (21.7%). Similarly, the spouses/partners of individuals from low-income and lower-middle class households also had the highest rate of employment status change (50%; see Figure 1). White families and families of color reported similar rates of employment status change (see Figure 2). Although the spouses/partners of parents of color appeared to have a higher rate of employment status change (36.7%) than their White counterparts (27.7%), the difference was not statistically significant.

#### Worked remotely

Table 2 presents the distribution of participants and their spouses/partners, when applicable, working remotely.

**Table 2.** Worked Remotely by Income and Race/Ethnicity.

	Self					
		Income Level			Race/Ethnicity	
	Overall (222)	Low (41)	Middle (52)	High (119)	White (144)	POC (78)
0% of the time	52 (23.4%)	19 (46.3%)	21 (40.4%)	9 (7.6%)	31 (21.5%)	21 (26.9%)
1%–24% of the time	15 (6.8%)	5 (12.2%)	3 (5.8%)	7 (5.9%)	10 (6.9%)	5 (6.4%)
25%–49% of the time	6 (2.7%)	2 (4.9%)	0 (0%)	3 (2.5%)	3 (2.1%)	3 (3.8%)
50%–74% of the time	17 (7.7%)	6 (14.6%)	3 (5.8%)	8 (6.7%)	7 (4.9%)	10 (12.8%)
75%–99% of the time	14 (6.3%)	1 (2.4%)	4 (7.7%)	8 (6.7%)	7 (4.9%)	7 (9%)
100% of the time	111 (50%)	7 (17.1%)	19 (36.5%)	80 (67.2%)	80 (55.6%)	31 (39.7%)
Other	7 (3.2%)	1 (2.4%)	2 (3.8%)	4 (3.4%)	6 (4.2%)	1 (1.3%)
	Spouse/Partner					
	Overall (188)	Low (20)	Middle (44)	High (116)	White (128)	POC (60)
0% of the time	57 (30.3%)	15 (75%)	20 (45.5%)	20 (17.2%)	33 (25.8%)	24 (40%)
1%–24% of the time	10 (5.3%)	1 (5%)	4 (9.1%)	4 (3.4%)	6 (4.7%)	4 (6.7%)
25%–49% of the time	9 (4.8%)	2 (10%)	3 (6.8%)	4 (3.4%)	7 (5.5%)	2 (3.3%)
50%–74% of the time	12 (6.4%)	1 (5%)	3 (6.8%)	8 (6.9%)	6 (4.7%)	6 (10%)
75%–99% of the time	11 (5.9%)	0 (0%)	3 (6.8%)	7 (6%)	8 (6.2%)	3 (5%)
100% of the time	82 (43.6%)	1 (5%)	9 (20.5%)	68 (58.6%)	61 (47.7%)	21 (35%)
Other	7 (3.7%)	0 (0%)	2 (4.5%)	5 (4.3%)	7 (5.5%)	0 (0%)

*Note*. Low = low-income and lower-middle class (≤ $50,000); Middle = middle class ($50,001–$100,000); High = upper-middle and high-income class (>$100,000); POC = people of color.

About half of the parents (self: 50%; spouse/partner: 43.6%) had switched to work remotely 100% of time since the outbreak of the COVID-19, but nearly 1 out 4 (self: 23.4%; spouse/partner: 30.3%) was unable to work remotely at all. Low-income and lower-middle class parents were significantly less likely to be able to work remotely than their middle-class, upper-middle, and high-income counterparts (self: χ^2^(12, *N*=212) = 54.94, *p*<.001, φ_*c*_=.36, large effect size; spouse/partner: χ^2^(12, *N*=180) = 47.35, *p*<.001, φ_*c*_=.36, large effect size). About half to three quarters (self: 46.3%; spouse/partner: 75%) of low-income and lower-middle class parents were unable to work remotely at all (0% of the time). In contrast, less than 18% of parents from high-income and upper-middle class were unable to switch to virtual work (self: 7.6%; spouse/partner: 17.2%).

POC parents (self: 39.7%; spouse: 35%), when compared to their White counterparts (self: 55.6%; spouse: 47.7%), were less likely to be able to switch to virtual work entirely (100% of the time). POC parents (self: 26.9%; spouse: 40%) also had a higher rate of not being able to work remotely at all (0% of the time) than that of White parents (self: 21.5%; spouse: 25.8%).

#### Clear structure and schedule at home to guide child’s learning

About half of the participants (49.5%) either agreed or strongly agreed that they had a clear structure and schedule at home to guide their children’s learning, while one-third of parents indicated that they did not have a clear structure and routines at home for their children (see Table 3). Families from different income levels appeared to perceive the level of structure and routines at home differently, χ^2^(8, *N*=186) = 18.21, *p*=.020, φ_*c*_=.22 (large effect size). Middle-class parents appeared to be less likely to feel there were clear structure and routines at home to guide their children’s learning (43.5%), when compared to parents of low-income and lower-middle class (49.5%) and those of upper-middle and high-income class (51.4%). In addition, more families of color either strongly agreed or agreed that they had clear structure and routines for their children at home (65.2%) than their White counterparts (41.9%),χ^2^(4, *N*=195) = 12.65, *p*=.012, φ_*c*_=.26 (large effect size).

**Table 3.** Clear Structure and Schedule at Home by Income and Race/Ethnicity.

		Income Level			Race/Ethnicity	
	Overall (186)	Low (33)	Middle (46)	High (107)	White (129)	POC (66)
Strongly disagree	14 (7.5%)	5 (15.2%)	1 (2.2%)	8 (7.5%)	9 (7%)	5 (7.6%)
Disagree	48 (25.8%)	5 (15.2%)	13 (28.3%)	30 (28%)	38 (29.5%)	11 (16.7%)
Neither agree or disagree	32 (17.2%)	6 (18.2%)	12 (26.1%)	14 (13.1%)	28 (21.7%)	7 (10.6%)
Agree	54 (29%)	10 (30.3%)	6 (13%)	38 (35.5%)	35 (27.1%)	22 (33.3%)
Strongly agree	38 (20.4%)	7 (21.2%)	14 (30.4%)	17 (15.9%)	19 (14.7%)	21 (31.8%)

*Note*. Low = low-income and lower-middle class (≤ $50,000); Middle = middle class ($50,001–$100,000); High = upper-middle and high-income class (>$100,000); POC = people of color.

#### Learning methods during school closure

Table 4 presents the data regarding learning methods that had been adopted by families since the outbreak of the COVID-19. The majority of families (83.2%) reported that their children continued their education via distance learning through their schools (e.g., web-based instructional methods, video conferencing, etc.). Distance learning through school was the primary and dominant learning method across income levels, and across racial/ethnic groups. However, children of upper-middle and high-income class were more likely to engage in distance learning through their schools than students of middle class and low-income and lower-middle class, χ^2^(6, *N*=187) = 12.79, *p*=.047, φ_*c*_=.19 (medium effect size).

**Table 4.** Learning Methods.

		Income Level			Race/Ethnicity	
	Overall (196)	Low (34)	Middle (46)	High (107)	White (130)	POC (66)
Distance learning (e.g., web-based instructional methods, video conferencing, etc.) through school	163 (83.2%)	24 (70.6%)	35 (76.1%)	96 (89.7%)	108 (83.1%)	55 (83.3%)
Homeschooling	10 (5.1%)	4 (11.8%)	5 (10.9%)	1 (0.9%)	2 (1.5%)	8 (12.1%)
Self-learning (e.g., children direct their own study)	6 (3.1%)	2 (5.9%)	1 (2.2%)	3 (2.8%)	5 (3.8%)	1 (1.5%)
Other	17 (8.7%)	4 (11.8%)	5 (10.9%)	7 (6.5%)	15 (11.5%)	2 (3%)

*Note*. Low = low-income and lower-middle class (≤ $50,000); Middle = middle class ($50,001–$100,000); High = upper-middle and high-income class (>$100,000); POC = people of color.

#### Consequences/problems associated with school closure as a result of the COVID-19 pandemic

**Figure 3.**
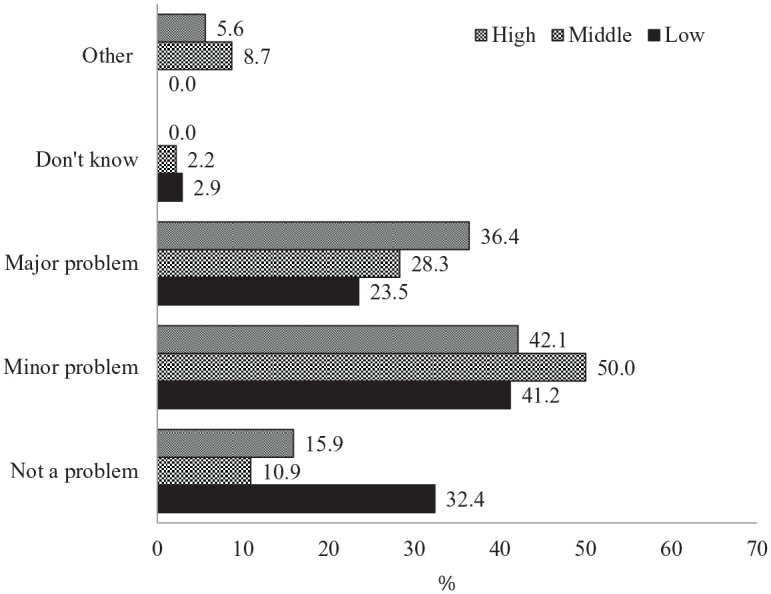
Extent to which child’s school closure had been a problem for family by income level. *Note*. Low = low-income and lower-middle class (≤ $50,000); Middle = middle class ($50,001–$100,000); High = upper-middle and high-income class (>$100,000).

**Figure 4.**
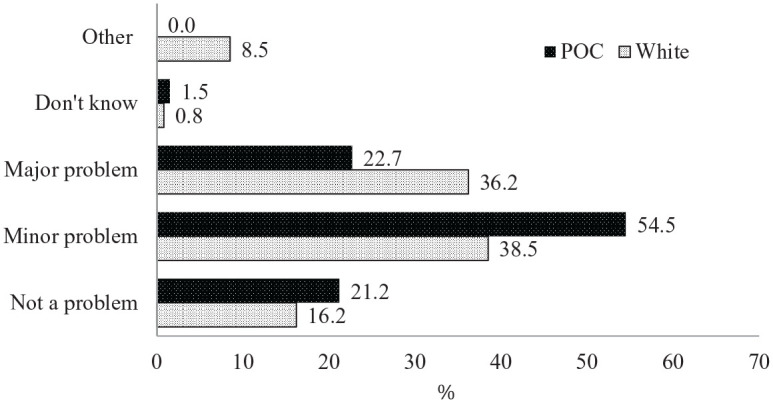
Extent to which child’s school closure had been a problem for family by race/ethnicity. *Note*. POC = people of color.

The majority of parents reported that school closures had been a major problem (31.6%), or a minor problem (43.9%) for their families, compared to those who said it was not a problem at all (17.9%). Upper-middle class and high-income families were more likely to report that school closure had been a major problem for them (36.4%) than middle-class families (28.3%) and families of low-income and lower-middle class (23.5%; see Figure 3). Moreover, a relatively higher percentage of low-income and lower-middle class families reported that school closure had not been a problem for them at all (32.4%) when compared to middle-class families (10.9%) and upper-middle and high-income families (15.9%). In addition, White families were more likely to report that school closures had been a major problem for them (36.2%) than families of color (22.7%; see Figure 4).

Table 5 presents the results regarding the specific consequences/problems associated with school closure as a result of the COVID-19 pandemic. Overall, more than half of the participants reported difficulty and stress over maintaining or creating structure and routines (70.1%), planning educational activities (61.9%), and planning physical activities (60.3%) for their children at home. Nearly one out of four parents reported that they had missed work (23.2%), and that they felt at risk of losing their job (20.1%). More than 10% of the participants had missed important appointments/events (18%), had lost pay or income (17.5%), and had incurred financial cost in excess of typical days (14.4%) and difficulty with arranging childcare (14.9%).

**Table 5.** Consequences/Problems as a Result of the COVID-19 Pandemic and School Closure.

		Income Level				Race/Ethnicity		
	Overall (194)	Low (33)	Middle (45)	High (107)	*p* value	White (129)	POC (65)	*p* value
Arranged childcare	29 (14.9%)	7 (21.2%)	7 (15.6%)	15 (14%)	^a^.610 ^b^.520 ^c^.321 ^d^.806	19 (14.7%)	10 (15.4%)	.904
Missed work	45 (23.2%)	10 (30.3%)	9 (20%)	26 (24.3%)	^a^.578^b^.295 ^c^.490 ^d^.565	26 (20.2%)	19 (29.2%)	.157
Child missed free or reduced-cost school meals	15 (7.7%)	7 (21.2%)	4 (8.9%)	4 (3.7%)	^a^.006**^b^.122^c^.001** ^d^.194	7 (5.4%)	8 (12.3%)	.090
Incurred financial cost in excess of typical days	28 (14.4%)	10 (30.3%)	9 (20%)	9 (8.4%)	^a^.005**^b^.295^c^.001** ^d^.044*	17 (13.2%)	11 (16.9%)	.484
Lost pay or income	34 (17.5%)	14 (42.4%)	6 (13.3%)	13 (12.1%)	^a^ <.001*** ^b^.004** ^c^<.001*** ^d^.840	22 (17.1%)	12 (18.5%)	.808
Missed appointment with potential financial impact	13 (6.7%)	5 (15.2%)	4 (8.9%)	4 (3.7%)	^a^.069† ^b^.392^c^.019* ^d^.194	5 (3.9%)	8 (12.3%)	.027*
Missed another kind of important appointment or event	35 (18%)	7 (21.2%)	11 (24.4%)	15 (14%)	^a^.264 ^b^.738 ^c^.321 ^d^.119	24 (18.6%)	11 (16.9%)	.774
Felt at risk of losing job	39 (20.1%)	13 (39.4%)	9 (20%)	16 (15%)	^a^.010* ^b^.060† ^c^.002** ^d^.444	27 (20.9%)	12 (18.5%)	.686
Child missed health services usually provided by school	17 (8.8%)	2 (6.1%)	8 (17.8%)	6 (5.6%)	^a^.043* ^b^.126 ^c^.922 ^d^.018*	11 (8.5%)	6 (9.2%)	.870
Felt stressed over planning educational activities for your child at home	120 (61.9%)	15 (45.5%)	27 (60%)	72 (67.3%)	^a^.076† ^b^.203 ^c^.024* ^d^.389	82 (63.6%)	38 (58.5%)	.490
Felt stressed over planning physical activities for your child at home	117 (60.3%)	15 (45.5%)	28 (62.2%)	69 (64.5%)	^a^.143 ^b^.141 ^c^.051† ^d^.791	82 (63.6%)	35 (53.8%)	.192
Felt stressed over maintaining or creating structure and routines for your child at home	136 (70.1%)	16 (48.5%)	33 (73.3%)	81 (75.7%)	^a^.010* ^b^.025* ^c^.003** ^d^.758	96 (74.4%)	40 (61.5%)	.064
Other	40 (20.6%)	6 (18.2%)	10 (22.2%)	23 (21.5%)	^a^.884 ^b^.627 ^c^.681 ^d^.868	29 (22.5%)	11 (16.9%)	.393

*Note*. Low (L) = low-income and lower-middle class (≤ $50,000); Middle (M) = middle class ($50,001–$100,000); High (H) = upper-middle and high-income class (>$100,000); POC = people of color.

a Significance level using chi-square test comparing L, M, and H classes.

b Significance level using chi-square test between L and M.

c Significance level using chi-square test between L and H.

d Significance level using chi-square test between M and H.

† *p* < .10. *p < .05. **p < .01. ***p < .001.

More than 40% of families from low-income and lower-middle class households had lost pay or income, which was significantly higher than those from middle class (13.3%), and upper-middle class and high-income households (12.1%), χ^2^(2, *N*=185) = 16.60, *p*<.001, φ_*c*_=.30 (medium effect size). Parents of low-income and lower-middle class were more likely to feel at risk of losing their job (39.4%) than their counterparts with higher household incomes, χ^2^(2, *N*=185) = 9.24, *p*=.010, φ_*c*_=.21 (medium effect size). More families of low-income and lower-middle class also experienced problems with incurred financial cost and children missing free or reduced-cost school meals, χ^2^(2, *N*=185) = 10.51, *p*=.005, φ_*c*_=.24 (medium effect size); and χ^2^(2, *N*=185) = 10.39, *p*=.006, φ_*c*_=.24 (medium effect size), respectively. Parents of low-income and lower-middle class were significantly more likely to miss such financially costing appointments than parents of upper-middle and high-income clas, χ^2^(1, *N*=140) = 5.46, *p*=.019, φ_*c*_=.20 (small effect size). Middle-class families were more likely to report that their children had missed health services usually provided by school, when compared to upper-middle and high-income families, χ^2^(1, *N*=152) = 5.61, *p*=.018, φ_*c*_=.19 (small effect size).

In contrast, a higher rate of upper-middle class and high-income families reported stress over maintaining or creating structure and routines for their children than that of low-income and lower-middle class families, χ^2^(1, *N*=140) = 5.11, *p*=.024, φ_*c*_=.19, (medium effect size). Middle class and higher income families were more likely to report stress over planning educational activities for their children at home (χ^2^(2, *N*=185) = 5.15, *p*=.076, φ_*c*_=.17, small effect size), than their low-income and lower-middle class counterparts, χ^2^(2, *N*=185) = 5.15, *p*=.076, φ_*c*_=.17, (small effect size).

Families of color, compared to their White counterparts, reported more problems with missing important appointments/events that had potential financial impact, χ^2^(2, *N*=194) = 4.92, *p*=.027, φ_*c*_=.16 (small effect size).

## Discussion

As the COVID-19 pandemic continued to be a global concern when this manuscript was being prepared in June 2020, the results of this study reflected the impact of the pandemic on families with school-aged children within the first three months of a rapid outbreak in the United States. Although the COVID-19 virus itself is supposed to be nondiscriminatory, our findings revealed inequitable consequences of the pandemic for low-income families and families of color. We observed that parents of low-income and lower-middle class households (≤US$50,000), as well as parents of color, experienced more adverse instrumental and financial hardships, such as reduced pay or income, furlough, and job loss or potential job loss. The findings are consistent with a recent report from the U.S. Department of Labor revealing that the historical layoffs due to the COVID-19 pandemic took the biggest toll on traditionally minoritized groups, including women, Blacks, Latinos, and the low-income workers (cited in Jones, 2020). In addition, we observed that low-income and lower-middle class parents, as well as parents of color, were less likely to have the privilege to work remotely from home, increasing their risk of exposure to the COVID-19 virus. A recent study (Hawkins, 2020) suggested that people of color are more vulnerable to COVID-19 infection due to the nature of their occupation and employment, as they are more likely to be considered “essential” or “frontline” workers in occupations with more exposure to infections and close proximity to others (e.g., food service, cleaning and building maintenance, retail and hospitality, warehouse work, public transit work, etc.).

The COVID-19 pandemic forced an unprecedented, massive school closure across the United States in the Spring of 2020. The majority of PreK–12 students across races and income levels continued their education through long-distance learning (e.g., web-based instructional methods, video conferencing, etc.) provided by their schools. However, we observed that students from low-income and lower-middle class households, compared to those from upper-middle and high-income families, appeared to have a lower rate of engagement in long-distance learning through school. Lack of resources, such as computers and Internet accessibility and stability, might have partially prevented students in low-income and lower-middle households from engaging in long-distance learning. To ensure equitable educational opportunities, schools need to further assess the barriers for students from low-income households to engage in long-distance learning and to provide needed equipment and support for them.

In addition, school meal programs are essential to many American families. We observed that low-income and lower-middle class families were more likely to be affected by their children missing free and reduced-price school meals due to the pandemic, when compared to higher income families. Communities and school need to work collaboratively to provide meals to students in need during school closures through different practices (e.g., curbside grab-and-go, delivery routes, mobile food pantries, etc.).

While students stayed home due to school closures in response to the pandemic, we observed that about one-third of families did not have a clear structure and schedule at home to support their children’s learning. Particularly, more middle-class parents appeared to struggle to create and maintain a clear structure and routines at home. It is possible that middle-class parents were cognizant of the importance of having a clear structure and routines at home but did not have the means for implementation. Schools may provide support through consultation to help parents design and implement customized structure and schedule plans for their children, while taking account of family structure, parent’s work schedule, and life circumstances.

Even though low-income and lower-middle class parents, as well as parents of color, experienced more instrumental and economic challenges, we observed that upper-middle and high-income parents were more likely to report that school closure had been a major problem for them. Particularly, parents of higher incomes and White parents were more likely to feel stressed over structuring home learning environments, and planning educational and physical activities at home for their children. The gap is most significant between parents of upper-middle and high-income class and those of low-income and lower-middle class. It is possible that upper-middle and high-income parents were more likely to work from home during the pandemic than low-income and lower-middle class parents; therefore, their immediate exposure to and association with their children’s learning at home resulted in additional responsibilities and perceived stress. It is also possible that low-income and lower-middle class parents were more likely to be overwhelmed by immediate financial challenges. They were more concerned about their family’s basic needs, such as food, clothes, and shelter, than structuring their children’s days and planning educational and physical activities at home for their children. Schools can play a pivotal role in providing needed psychological and instrumental support based on families’ specific needs.

This study has some limitations. Because of the cross-sectional nature of this observational study, causation can never be exactly known. It is possible that the racial and income differences observed in this study might be partially attributed to other social and political events and conflicts (e.g., police brutality, racism, Black Lives Matter Movement) that were simultaneously occurring in the U.S. in the era of the COVID-19 pandemic (Thomeer et al., 2020). More research is needed to understand the complexity of the dynamic interplay of social, political, and economic factors in families’ lives during a pandemic. The majority of the participants were White females from higher income clusters. Parents from low-income and middle classes were disproportionately underrepresented in the sample. Although participants resided in 26 states in the United States, more than half majority were from New York State. Our online recruitment efforts might not reach families that had zero or limited access to the Internet or the sites where our online messages were posted. Therefore, the sample might not represent the entire U.S. population. However, we did not intend to conduct this study as a population research to understand the overall impact of the COVID-19 on families, but to focus on the roles of income level and race/ethnicity in the experiences of families with school-aged children during the pandemic. Our findings highlight the importance of evaluating the instrumental and psychosocial impact of the pandemic on families. Moreover, as we observed inequitable outcomes of the pandemic for low-income and racially minority families, schools and communities should further assess and address the specific challenges and difficulties facing diverse families in order to provide needed support for them.
